# Artefacts in Volume Data Generated with High Resolution Episcopic Microscopy (HREM)

**DOI:** 10.3390/biomedicines9111711

**Published:** 2021-11-18

**Authors:** Lukas F. Reissig, Stefan H. Geyer, Julia Rose, Fabrice Prin, Robert Wilson, Dorota Szumska, Antonella Galli, Catherine Tudor, Jacqueline K. White, Tim J. Mohun, Wolfgang J. Weninger

**Affiliations:** 1 Division of Anatomy, Center for Anatomy and Cell Biology, Medical University of Vienna, 1090 Vienna, Austria; stefan.geyer@meduniwien.ac.at (S.H.G.); julia.rose.meduni@gmail.com (J.R.); wolfgang.weninger@meduniwien.ac.at (W.J.W.); 2 Crick Advanced Light Microscopy Facility, The Francis Crick Institute, London NW1 1AT, UK; Fabrice.Prin@crick.ac.uk; 3 The Francis Crick Institute Mill Hill Laboratory, London NW7 1AA, UK; rwilson@ebi.ac.uk (R.W.); tim.mohun@gmail.com (T.J.M.); 4 Wellcome Trust Centre for Human Genetics, Oxford OX3 7BN, UK; dorota.szumska@cardiov.ox.ac.uk; 5 Wellcome Trust Sanger Institute, Hinxton, Cambridge CB10 1RQ, UK; antonella.galli@abcam.com (A.G.); catherine.jones@sanger.ac.uk (C.T.); jacqui.white@jax.org (J.K.W.)

**Keywords:** HREM, artefacts, block face imaging, histology, embryo, genetically engineered, mouse embryo

## Abstract

High resolution episcopic microscopy (HREM) produces digital volume data by physically sectioning histologically processed specimens, while capturing images of the subsequently exposed block faces. Our study aims to systematically define the spectrum of typical artefacts inherent to HREM data and to research their effect on the interpretation of the phenotype of wildtype and mutant mouse embryos. A total of 607 (198 wildtypes, 409 mutants) HREM data sets of mouse embryos harvested at embryonic day (E) 14.5 were systematically and comprehensively examined. The specimens had been processed according to essentially identical protocols. Each data set comprised 2000 to 4000 single digital images. Voxel dimensions were 3 × 3 × 3 µm^3^. Using 3D volume models and virtual resections, we identified a number of characteristic artefacts and grouped them according to their most likely causality. Furthermore, we highlight those that affect the interpretation of embryo data and provide examples for artefacts mimicking tissue defects and structural pathologies. Our results aid in optimizing specimen preparation and data generation, are vital for the correct interpretation of HREM data and allow distinguishing tissue defects and pathologies from harmless artificial alterations. In particular, they enable correct diagnosis of pathologies in mouse embryos serving as models for deciphering the mechanisms of developmental disorders.

## 1. Introduction

High resolution episcopic microscopy (HREM) is a technique for generating digital volume data of organic material with volumes of up to 8 × 10 × 15 mm^3^ in typical numeric isotropic resolutions of 1–5 µm [1]. The method already proved to work with various tissues harvested from adult biomedical models, humans and plants, as well as paper, synthetic skin substitute material and others [2,3,4,5]. Yet, its chief domains are the visualization of skin [6,7,8] and the phenotyping of embryos of biomedical models, with a focus on the mouse [9]. In the latter, the high quality of HREM data permits detailed visualization of tissue architecture, gross morphology and organ topology in the context of all organ systems of early to late embryos [1,10].

Preparing tissues for HREM imaging requires their fixation, dehydration and embedding in eosin dyed methacrylate resin (JB4, Polysciences). This is similar to preparing specimens for traditional histology and consequently introduces similar artificial changes, such as inhomogeneous shrinkage or swelling of tissues and cells, detachment of epithelia from the underlying tissue, formation of vacuoles during fixation or incorporation of undissolved stain particles or air bubbles [11,12,13]. Once hardened, the resin blocks are mounted on an HREM apparatus where they become physically sectioned, while digital images are captured from each freshly exposed block surface. The sections themselves are usually discarded. Therefore, in contrast to images captured from subsequent physical sections, series of HREM images are inherently aligned and do not exhibit non-affine distortions or tissue expansions introduced by sectioning and section stretching and mounting [14]. They can be immediately virtually stacked, converted to digital volume data and visualized with simple volume rendering algorithms. The precise alignment of the section images and their excellent contrast and resolution permit high detail morphological and topological analyses by using simple virtual resections. Metric analyses are possible after producing surface rendered computer representations [1,2,6,10].

The single steps required for producing HREM data, such as sample harvesting, fixation, dehydration, contrasting, embedding and data generation introduce artefacts. They often obscure information and even worse, lead to false interpretation of the HREM data. Consequently, this may result in false assessment of the morphological information and false diagnoses of pathologies [13]. It is therefore of utmost importance to systematically identify and define artefacts introduced during HREM data generation and to discuss their specific effects on HREM data appearance and quality as well as their potential for misinterpretations.

For its obvious advantages, HREM was selected in the “Deciphering the Mechanisms of Developmental Disorders” (DMDD) program for scoring the phenotype of genetically engineered mouse embryos harvested at embryonic day (E) 14.5. DMDD was a strategic program founded by the Wellcome Trust and associated with the International Mouse Phenotyping Consortium [15]. It aimed at characterizing embryos of systematically produced mouse lines with gene deletions that produce pre- or perinatally lethal homozygous offspring [16,17]. A total of 240 single knockout lines were produced, and embryos, together with their placentae were collected and processed [18]. HREM data were created from mutants of 81 knockout (KO) lines, which produced offspring that survived until E14.5. The data were used for comprehensive phenotyping according to a standardized protocol [10]. Abnormalities in morphology, topology and architecture of organs, anatomic structures and tissues were carefully annotated using the mammalian phenotype (MP) ontology (http://www.informatics.jax.org/vocab/mp_ontology, last access on 24 October 2021). The annotated data are published on the DMDD homepage (dmdd.org.uk) which is hosted by the Francis Crick Institute and integrated into the Mouse Genome Informatics database (MGI, http://www.informatics.jax.org/, last access on 24 October 2021) [19]. Phenotyping mutants was flanked by producing anatomic reference data based on wildtype embryos to allow scientists to correctly identify phenotypic abnormalities and to describe anatomical variations and developmental peculiarities [20,21,22]. In sum, DMDD produced a total of over 600 HREM data sets of E14.5 embryos under largely standardized conditions. These HREM data provide a unique opportunity to systematically study the appearance and frequency of typical artefacts affecting HREM data in general and HREM data of mouse embryos in particular.

We therefore decided to make use of this unique resource and designed a study, aiming at providing comprehensive descriptions and analyses of artefacts, which HREM data typically comprise. This information will assist the steadily growing community of HREM users in distinguishing artefacts from structure and tissue abnormalities and help them to optimize the HREM protocols for examining various types of specimens and materials. In particular, it will assist all scientists working with data of DMDD mutants and with mouse embryo data produced in stand-alone projects to correctly interpret and classify observed phenotypes.

## 2. Materials and Methods

### 2.1. Embryos

We systematically reviewed 607 HREM data sets from embryos harvested at embryonic day (E) 14.5. Out of all the embryos, 409 were mutants, derived from a total of 74 single knockout lines that produced subviable or prenatally lethal offspring, which survived at least until E14.5, and 198 were wildtype controls. All embryos were bred on the C57BL/6N background at the Wellcome Trust Sanger Institute in scope of the deciphering the mechanisms of developmental disorders (DMDD) program [16,17].

### 2.2. Embryo Harvesting

Embryos were exposed by opening the abdominal cavity and the uterine horns of the dams. Together with their placentae they were removed and placed in 37 °C phosphate buffered saline (PBS). Using two-pointed forceps the amniotic sacs were opened, and the umbilical cords were cut. Then, the external morphology of the embryos was systematically checked for gross anomalies under a dissection microscope and the embryos and placentae were separately placed in tubes and fixed in Bouin’s fixative for at least 24 h.

### 2.3. Specimen Preparation and Embedding

Fixed specimens were dehydrated, infiltrated and embedded in eosin dyed methacrylate resin. For dehydration, the specimens were transferred into an ascending methanol series (10% increments until 90%, then 95% and 100%, 1 h each, constant gentle rocking). Following dehydration, the specimens were infiltrated with JB-4 infiltration solution (Polysciences, Inc., Warrington, PA, USA) for 72 h and embedded in JB-4 embedding solution. Eosin B (Sigma-Aldrich, St. Louis, MO, USA) (0.275 g/100 mL) and acridine orange (Sigma-Aldrich, St. Louis, MO, USA) (0.055 g/100 mL) were added to both solutions [9].

For embedding, the specimens were transferred from the infiltration solution into embedding molds filled with embedding solution. Embryos were placed, head down and carefully positioned with their cranio-caudal axis perpendicular to the future block surface as soon as the viscosity of the embedding solution started to increase. For this, forceps or a blunt needle were used. As soon as the embryos were fixed by the hardening resin, block holders were placed on the embedding molds. Then, the molds were fully filled and sealed air proof using mineral oil to prevent oxygen to affect polymerization. After polymerization at room temperature for 12–24 h, the resin blocks were baked at 90 °C for 24–48 h to speed up the hardening process [9,23].

### 2.4. HREM Data Generation

For HREM data generation an HREM-prototype based on a Leica SM2500 microtome as well as a commercial Optical-HREM apparatus (Indigo Scientific Ltd., Baldock, UK) was used according to the manual and established protocols [2,9,10,17].

### 2.5. Data Processing and Analysis

Images were captured as 12-bit TIFF images. In order to cope with the different grayscale ranges depending on the operator or the amount of dye included in the sample, routines running in Photoshop 6 (Adobe Inc., San José, CA, USA) were used to optimize contrasts and brightness. In the same routine the images were also mildly sharpened, cropped, scaled to match cubic voxels of 3 × 3 × 3 µm^3^ and converted to 8-bit TIFF images. The TIFF images were virtually stacked and converted to JPEG images (quality 80%). The stacks were scaled to 50% to produce data small enough to assist data handling during phenotype screening and annotation.

Data visualization and analysis was performed on MacPro computers (Apple Inc., Cupertino, CA, USA) (64 GB RAM, MacOS X) with 32′ UHD monitors (BenQ, Taipeh, Taiwan), operating the Osirix software (Version 10, Pixmeo Sàrl, 1233 Bernex, Switzerland, open-source software; www.osirixviewer.com, last access on 24 October 2021) and on high end PCs (128 GB RAM, Windows 10 (Microsoft Corporation, Albuquerque, NM, USA) operating the Amira software package (Version 6.7, Thermo Fisher Scientific, Waltham, MA, USA).

Data collection and statistical analysis were performed using Microsoft Excel (Microsoft Office professional 2016, Microsoft Corporation, Albuquerque, NM, USA) and SPSS (IBM SPSS Statistics version 24; IBM Corporation, Armonk, NY, USA). To evaluate a potential effect of the genotype on the artefact incidence, group comparisons were performed using the χ^2^ test or Fisher’s exact test if the expected cell count was less than five. *p*-values less than 0.05 were considered statistically significant.

## 3. Results

**Figure 1:**
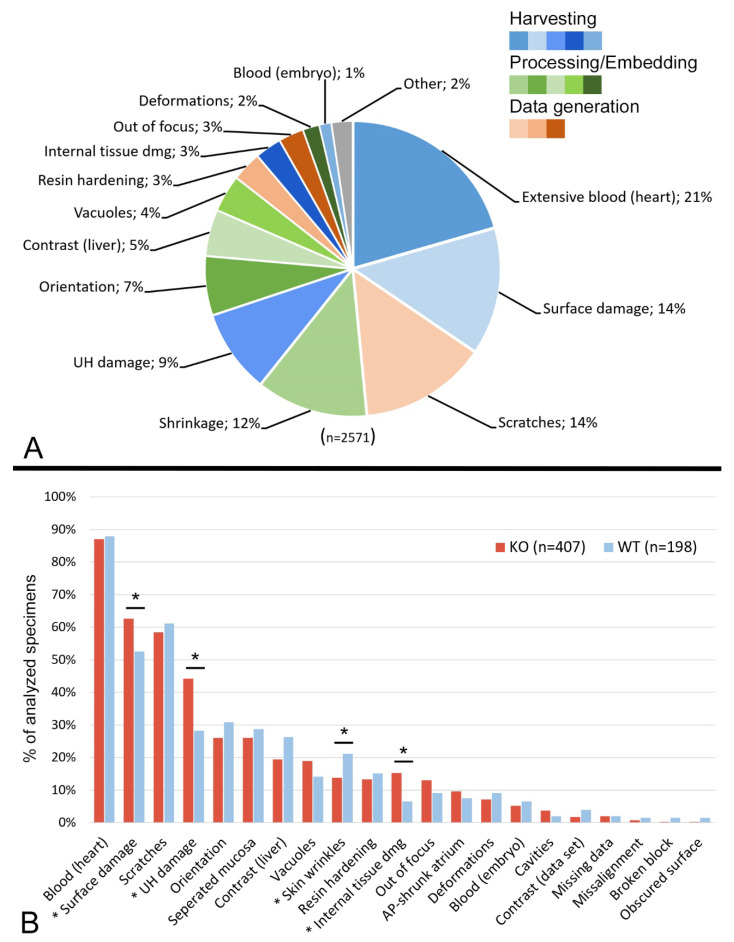
Frequency of artefacts. (**A**) Pie chart of artefact types relative to the total number of described artefacts. Harvesting artefacts in blue, processing/embedding artefacts in green, data generation artefacts in orange. “Shrinkage” includes extensive skin wrinkles, shrunk cardiac atrium and separated stomach mucosa; “UH damage” includes damaged and removed umbilical hernia and damaged intestinal slings; “Internal tissue damage” includes tissue damage with or without surface damage. (**B**) Bar graph of artefact types relative to total number of analyzed specimens, comparing wild type and mutants. Asterisk indicates artefact type with significant difference between KO and WT embryos (* *p* < 0.05). Abbreviations: AP: anterior-posterior; KO: knockout; WT: wildtype; UH: umbilical hernia.

Artefacts were observed in all HREM data examined. Most of them had obvious causalities. For classification, we grouped these artefacts according to their most likely reason and distinguished specimen harvesting, specimen processing and data generation artefacts (Figure 1, Supplementary Table S1). The majority of the artefacts appeared to be unrelated to the genotype, except for physical damages and shrinkages (Figure 1).

### 3.1. Specimen Harvesting Artefacts

**Figure 2:**
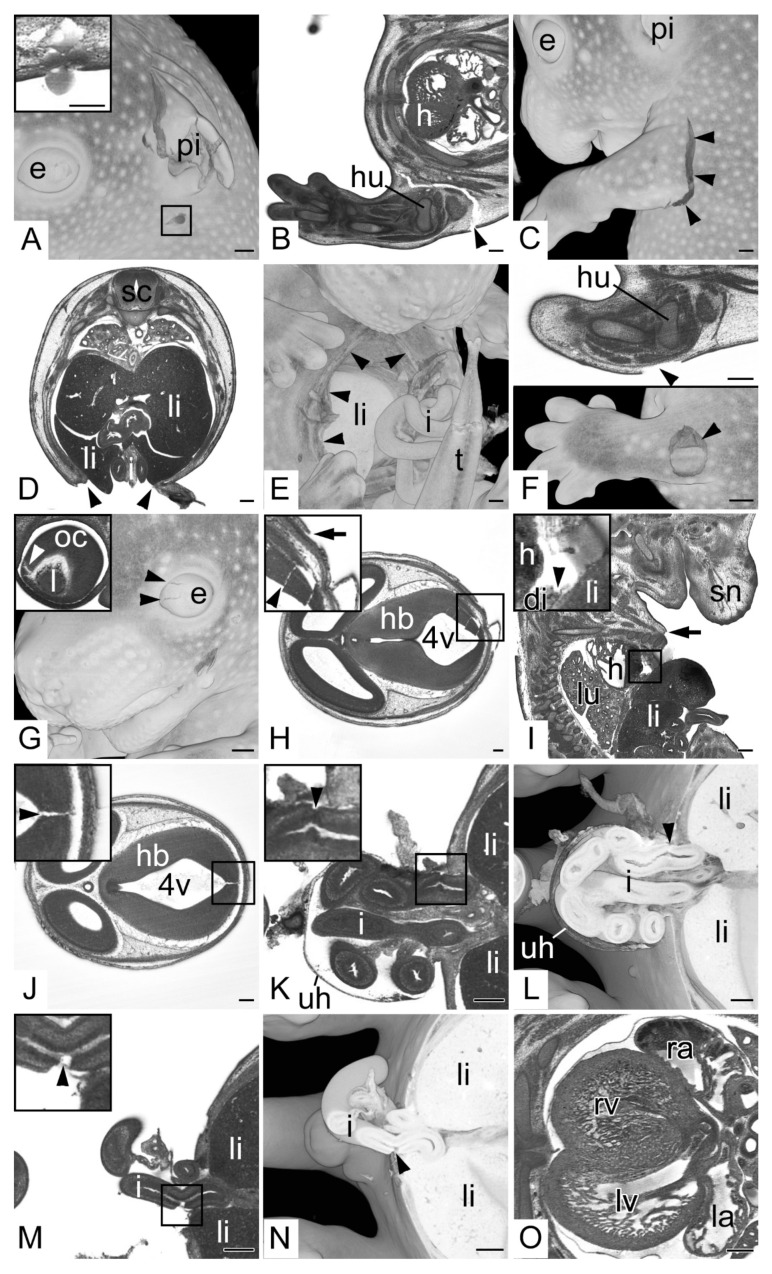
Specimen harvesting artefacts. (**A**) Semitransparent volume model of the head from the left. Pinna (pi) broken. Box indicating puncture damage. Inlay: transverse HREM section showing puncture damage. (**B**,**C**) Torn upper left extremity (arrow heads), (**B**) transverse HREM section, ventral to the left (**C**) 3D model from the left. (**D**,**E**) Ruptured ventral body wall (arrow heads) liver (li) protruding from abdominal cavity, (**D**) transverse HREM section, ventral on bottom, (**E**) semitransparent volume model from ventral. Note the broken tip of the tail (t). (**F**) Circumscribed damage of the skin of the left arm (arrow head), transverse HREM section on top, semitransparent volume model on bottom. Note the underlying muscle visible in the 3D model. (**G**) Damaged eyeball (e) (black arrow heads), semitransparent volume model from the left. Inlay: 2D section showing teared optic cup (oc) (white arrow head). (**H**) Damage of hindbrain (hb) (arrow head) in combination with surface damage (arrow), transverse HREM section ventral to the left. (**I**) Damage of the diaphragm (di) combined with ruptured body wall (arrow), sagittal resection ventral to the right. (**J**) Hindbrain (hb) defect (arrow head) in embryo with intact surface, transverse HREM section, ventral to the left. (**K**,**L**) Torn intestine (i) (arrow head) in embryo with intact wall of umbilical hernia (uh). (**K**) Transverse HREM section, ventral to the left. (**L**) Semitransparent volume model transected at height of (**K**). (**M**,**N**) Torn intestine (i) (arrow head) in embryo with damaged wall of umbilical hernia. (**M**) Transverse HREM section, ventral to the left. (**N**) Semitransparent volume model transected at height of (**M**). (**O**) Heart partly filled with blood. Note the difference in visibility of the internal structures of the blood-filled right ventricle (rv) and right atrium (ra) compared to the empty left ventricle and atrium (lv,la). Abbreviations: 4v: 4th ventricle, di: diaphragm, e: eye, h: heart, hb: hindbrain, hu: humerus, i: intestine, l: lens, la: left atrium, li: liver, lv: left ventricle, oc: optic cup, pi: pinna, ra: right atrium, rv: right ventricle, sc: spinal cord, sn: snout, t: tail, uh: umbilical hernia. When not stated otherwise, box indicates magnification in inlay. Scale bars = 250 µm.

Opaque volume models revealed that 359 embryos (59.1% total; 62.3% KO; 52.5% WT) had defects on their surface. This included 84 embryos (13.8% total; 15.4% KO; 10.6% WT) that showed deep tissue damage of the head and/or a torn or broken pinna (Figure 2A), 152 embryos (25.0% total; 26.4% KO; 22.2% WT) that had one or more limbs broken or torn from the body (Figure 2B,C) and 56 embryos (9.2% total; 9.5% KO; 8.6% WT) that showed extensive defects of the body wall (Figure 2D,E). In the rest of the embryos, that did not feature extensive head, limb or body wall damage, the skin was torn and partly ripped from the subcutis or the underlying superficial muscles at singular, small and circumscribed areas, the size of needle tips or slightly larger (Figure 2F). In 41 embryos (6.8% total; 8.3% KO; 3.5% WT) surface defects were associated with damaged internal tissues, including damages of the lens and optic cup, brain cortex and liver, and even with the rupture of the diaphragm and the descending aorta (Figure 2G–I)

In 34 embryos (5.6% total; 6.8% KO; 3.0% WT) internal structures, such as brain cortex or intestinal slings were damaged although the skin and the membranes of the umbilical hernia were intact (Figure 2J–L).

In 192 embryos (31.6% total; 34.7% KO; 25.3% WT) the wall of the physiological umbilical hernia was damaged. In 38 embryos (6.3% total; 7.8% KO; 3.0% WT) this was associated with ruptured intestinal slings (Figure 2M,N). In 6 (1.0% total; 1.5% KO; 0.0% WT) the hernia and its content were entirely removed, and the umbilical annulus was wide open. In 34 embryos (5.6% total; 5.1% KO; 6.6% WT) virtual sections revealed considerable amounts of free blood in the umbilical hernia and around the umbilical annulus, even extending into the embryo body.

All embryos showed blood clots in all components of their cardiovascular systems. Additionally, all had their veins filled more extensively than the arteries. In none of the embryos were the cardiac chambers completely free of blood. In 348 embryos (57.3% total; 58.4% KO; 55.1% WT) the blood even filled 50% to 75% and in an additional 180 (29.7% total; 28.1% KO; 32.8% WT) more than 75% of the cavity of the cardiac ventricles and atria (Figure 2O).

### 3.2. Specimen Processing and Embedding

**Figure 3:**
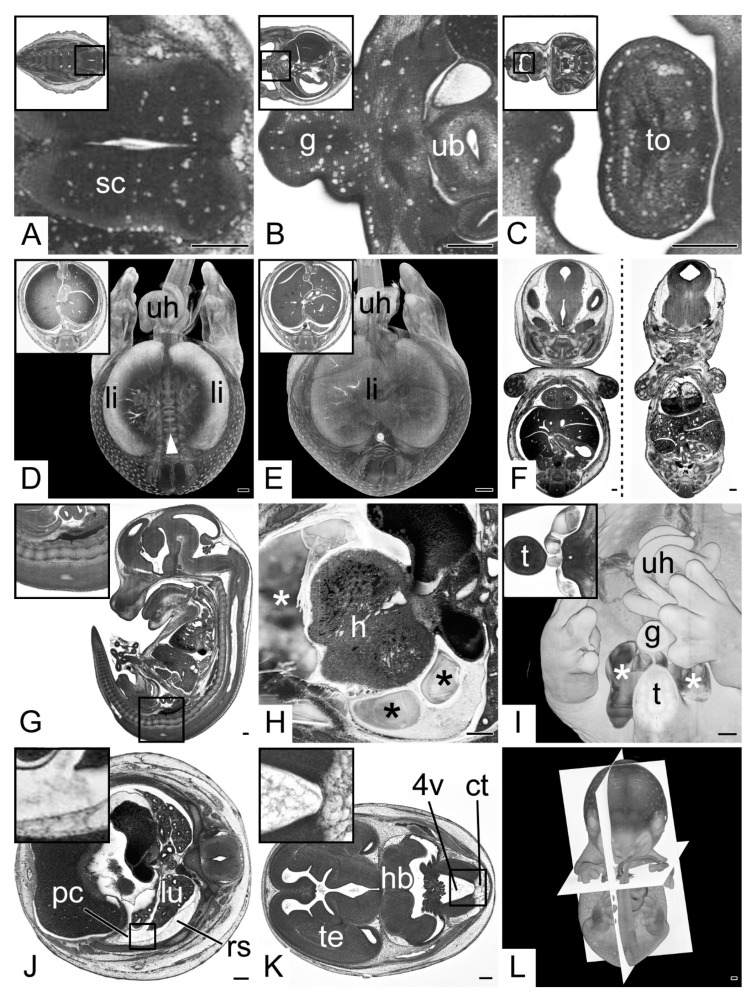
Specimen processing and embedding artefacts. (**A**–**C**) Vacuoles in various tissues. Transverse HREM sections, ventral to the left. Inlays: overview. (**A**) Caudal segment of spinal cord (sc). (**B**) Genital region (g). (**C**) Tongue (to). Note the vacuoles following the tissue border between mucosa and muscle tissue. (**D**,**E**) Low tissue contrast inside liver (li). Semitransparent volume model transected at the height of the umbilical hernia (uh). Inlays: Transverse HREM sections. (**D**) Due to the low contrast of the liver, its center is not rendered and the underlying vertebral column (white arrow head) is visible. (**E**) control. (**F**) Lateral deformation, coronal resections. Deformed embryo on the right showing lateral compression and flattening. Control on the left. (**G**) “Pseudolordosis” of the sacral spine, sagittal resection, ventral to the left. (**H**,**I**) Cavities (*) in resin block (**H**) Cavities (*) inside the pericardial sac near the heart (h). Transverse HREM section, ventral to the left. (**I**) Cavities (*) outside the embryo body, near the base of the tail (t). A 3D model from ventral. Inlay: Transverse HREM section. (**J**,**K**) Debris inside body cavities. Transverse HREM sections, ventral to the left. (**J**) Debris inside pleural cavity (pc). Compare the adjacent retropleural space (rs). (**K**) Debris in 4th brain ventricle (4v) compare the leptomeningeal connective tissue (ct). In addition, note the appearance of the telencephalic (te) ventricles. (**L**) Poorly aligned embryo. A 3D model strictly from ventral. Orientation planes as defined by the embedding block in white. Abbreviations: 4v: 4th ventricle, ct: leptomeningeal connective tissue, g: genitalia, h: heart, hb: hindbrain, pc: pleural cavity, rs: retropleural space, sc: spinal cord, t: tail, te: telencephalon, to: tongue, ub: urinary bladder, uh: umbilical hernia. When not stated otherwise, box indicates magnification in inlay. Scale bars = 250 µm.

In HREM sections of 105 embryos (17.3% total; 18.8% KO; 14.1% WT) the dense tissues of organs had a spongy appearance, featuring multiple, small vacuoles with an average diameter of approximately 20 µm (Figure 3A–C). In 11 embryos (1.8% total; 2.4% KO; 0.5% WT) such vacuoles were located in all organs in extensive numbers. In 25 embryos (4.1% total; 3.4% KO; 5.6% WT) they were only sparsely distributed in some organs. In 69 embryos (11.4% total; 13.0% KO; 8.1% WT) a small number appeared almost exclusively in the caudal segments of the central nervous system.

The HREM data of 131 embryos (21.6% total; 19.3% KO; 26.3% WT) showed poor tissue contrasts in the center of the liver (Figure 3D,E).

Eleven embryos (1.8% total; 1.7% KO; 2.0% WT) had their cranio-caudal axis shortened as if longitudinally compressed. Thirty-six embryos (5.9% total; 5.4% KO; 7.1% WT) had small biparietal diameters and appeared as if their bodies were laterally compressed (Figure 3F).

The sagittal virtual resections showed that all embryos had an indentation of the sacral spine resembling an unnatural lordosis (Figure 3G).

Virtual volume models of 19 specimens showed irregularly shaped hollow spaces inside the resin blocks (3.1% total; 3.7% KO; 2.0% WT). In 12 (2% total; 2.4% KO; 1.0% WT) they were situated inside the embryo, chiefly in the subcutis of the caudal body parts or inside the pericardial cavity (Figure 3H). In five (0.8% total; 0.7% KO; 1.0% WT) they were located outside the embryo, but near its surface with a close association to the root of the tail (Figure 3I). In two cases (0.3% total; 0.5% KO; 0.0% WT) hollow spaces were located in and outside the embryo body.

All embryos showed loose debris-like material scattered inside body cavities, such as the pericardial, peritoneal and pleural cavity (Figure 3J). Strikingly, such debris also occurred inside the brain ventricles, which, in addition, contained material resembling the appearance of histologically processed gelatinous fluids (Figure 3K). When distributed homogeneously this debris showed close similarity with loose adjacent tissues such as edematous retropleural tissue or leptomeningeal connective tissue.

The longitudinal axes of the models of 131 embryos (21.6% total; 20.3% KO; 24.2% WT) were tilted for more than five degrees in the coronary and/or sagittal plane in respect to the block surface. In 91 of those embryos (15.0% total; 14.4% KO; 16.2% WT) the deviation of the axis was larger than ten degrees in one direction. The models of 36 (5.9% total; 5.6% KO; 6.6% WT) embryos showed a rotation of more than five degrees around the longitudinal axis; in 26 (4.3% total; 3.4% KO; 6.1% WT) of them the rotation was greater than ten degrees (Figure 3L).

**Figure 4:**
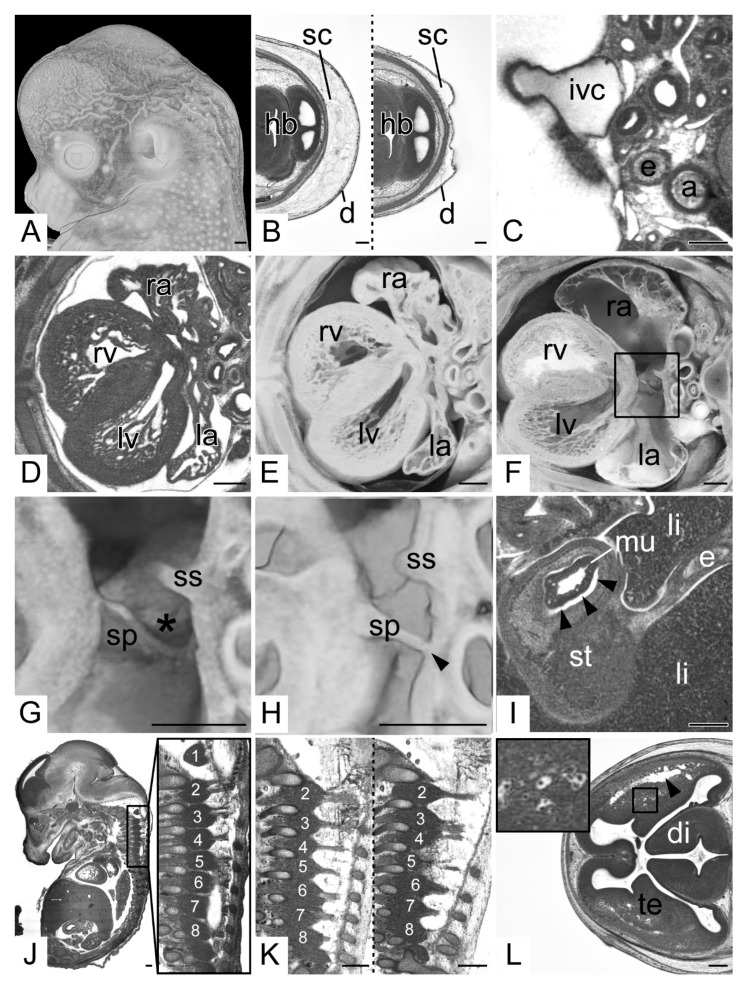
Shrinkage artefacts. (**A**) Wrinkled skin. Volume model from left. (**B**) Thickness of the subcutis (sc). Transverse HREM sections, ventral to the left. (**C**) Cross section of the inferior vena cava (ivc). Compare shape to aorta (a). Transverse HREM section, ventral to the left. (**D**–**F**) Atrial appendages. (**D**) Transverse HREM section, ventral to the left. (**E**) Transected 3D model. (**F**) Control. Note the wall shrinkages and cavity dimensions (ra, la). (**G**,**H**) Foramen ovale (asterisk). Transected semitransparent volume models. (**G**) is a magnification of (**F**) and serves as control. (**H**) Septum primum (sp) touching the dorsal atrial wall (arrow head). The foramen ovale appears as if closed. (**I**) Artificial detachment of stomach (st) mucosa (mu). Transverse HREM section (**J**,**K**) Cervical spinal ganglia (1–8). Sagittal resections, ventral to the left. (**J**) Demarcated ganglion material. (**K**) Connected ganglia (2–8) in a wildtype (left side) and a 4933434E20Rik mutant (right side). Note the separated dorsal roots in the wildtype and the mingled dorsal roots of 3 and 4 in the mutant. (**L**) Spongy appearance of brain tissue. Elongated cavities profound to the superolateral cerebral cortex (arrow head) and cavities surrounding intracerebral blood vessels. Inlay: Magnification of cavities around blood vessels. Abbreviations: 1–8: cervical dorsal root ganglion 1–8, a: aorta, d: dermis, di: diencephalon, e: esophagus, hb: hindbrain, ivc: inf vena cava, la: left atrium, li: liver, lv: left ventricle, mu: mucosa, ra: right atrium, rv: right ventricle, sc: subcutis, sp: septum primum, ss: septum secundum, st: stomach, te: telencephalon. Scale bars = 250 µm.

In all data, features linked to tissue shrinkages were clearly visible. The skin of all embryos showed wrinkles. In 98 specimens (16.1% total; 13.7% KO; 21.2% WT) the depth of the wrinkles exceeded 150 µm (Figure 4A). The thickness of the subcutis varied greatly (Figure 4B). Muscles, organs and other anatomical features also showed signs of shrinkages. The most prominent examples for artificial alterations resulting from them are: (1) the shape of the veins: they did not appear as roundish vessels, but always had an irregular perimeter, no matter if free of or filled with blood (Figure 4C); (2) the appearance of the pericardium and the central tendon of the diaphragm: both showed deep irregular wrinkles; (3) the shape of the atria: in particular the atrial appendices were crumpled to various degrees (Figure 4D,E). In 54 embryos (8.9% total; 9.5% KO; 7.6% WT) the antero-posterior extension of the atria was reduced to feature the free rim of the septum primum directly touching the dorsal atrial wall. Hence, 3D models of these specimens did not feature a foramen ovale but completely separated atria instead (Figure 4G,H); (4) the irregular shape of the optic cups; (5) the detached intestinal mucosa: all embryos showed detachments of mucosa, submucosa and muscle layers in segments of the stomach and intestine to various degrees, e.g., the mucosa of the pyloric segment of the stomach was detached in 163 (26.9% total; 25.9% KO; 28.8% WT). However, only in 48 embryos (7.9% total; 7.1% KO; 9.6% WT) (Figure 4I) it was detached from all segments; (6) the position of the spinal ganglia: often the material between subsequent ipsilateral spinal ganglia was reduced in a way that was is barely discernible. The ganglia therefore appeared as if fused (Figure 4J,K); (7) the texture of the brain tissue: especially at the borders between central gray and white matter, elongated gaps appeared and the vessels often lay in cavities, which resulted in a spongy appearance of the brain tissue (Figure 4L).

### 3.3. Data Generation

**Figure 5:**
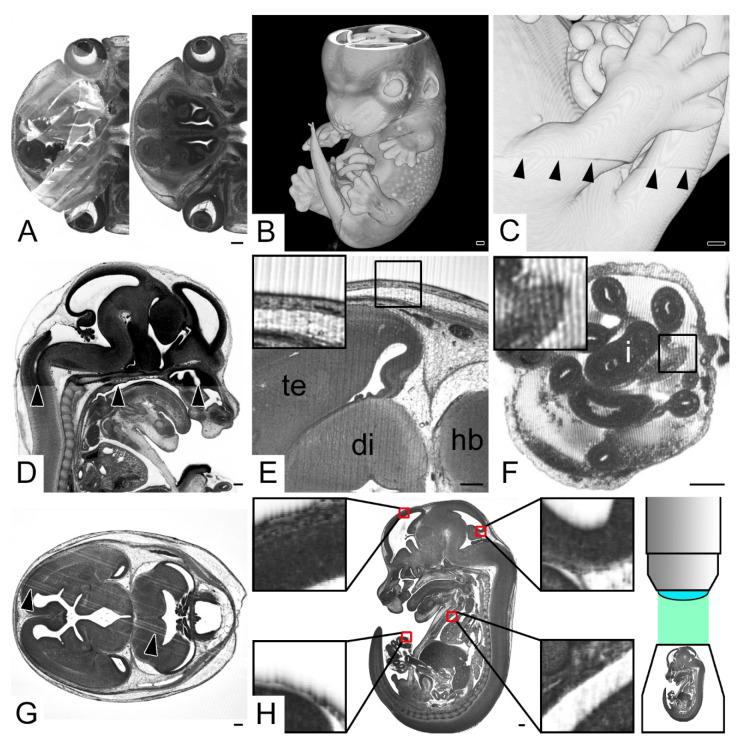
Data generation artefacts. (**A**) Original HREM section image partly obscured by unremoved section. (**B**) Volume model of embryo missing images from top of the head (**C**) Misalignment between stacks of consecutive HREM images (arrow heads). A 3D model from right. (**D**) Change of image contrast (arrow heads) at the level of the hard palate. Sagittal resection, ventral to the right. (**E**,**F**) Straight (**E**) and wave-like (**F**) lines perpendicular to the cutting direction in original HREM images. (**G**) Scratches in original HREM sections running in parallel to the cutting direction. (**H**) “Bleeding through”. Sagittal resection, ventral to the left. Inlays show magnifications of areas inside the red boxes. The cranial borders of darkly stained structures appear as fading into cavities in the direction of the image capturing optics. Note the scheme of the optical setup indicating that the embryo is imaged from cranial to caudal. Abbreviations: di: diencephalon, hb: hindbrain, i: intestine, te: telencephalon. Scale bars = 250 µm.

Four HREM data (0.7% total; 0.2% KO; 1.5% WT) had a clearly visible crack crossing the block and parts of the embryo. In another four (0.7% total; 0.2% KO; 1.5% WT) several original HREM images appeared as partly obscured by an indifferent whitish fog. The obvious reason was a recently cut section, which loosely stuck to the freshly exposed block face during image capturing (Figure 5A).

Twelve HREM data sets (2.0% total; 2.0% KO; 2.0% WT) missed images. From those, six data sets (1.0% total; 1.2% KO; 0.5% WT) missed a significant number of section images from the top of the image stack (Figure 5B). One data set (0.2% total; 0.2% KO; 0.0% WT) missed a group of sections from the middle of the image stack cutting through the head and neck of the embryo. Four data sets (0.7% total; 0.2% KO; 1.5% WT) missed a large number of section images in the bottom of the image stack.

In one data set (0.2% total; 0.2% KO; 0.0% WT) the back of the embryo was out of the field of view and six data sets (1.0% total; 0.7% KO; 1.5% WT) appeared to have misaligned section images (Figure 5C). Fifteen HREM data sets (2.5% total; 1.7% KO; 4.0% WT) showed groups of a few hundred subsequent sections, in which contrast and brightness differed dramatically, compared to the rest of the image stack (Figure 5D). Seventy-one HREM data sets (11.7% total; 13.0% KO; 9.1% WT) held single images or groups of subsequent sections which were slightly out of focus.

All images of 84 HREM data sets (13.8% total; 13.2% KO; 15.2% WT) showed smooth, ripple-like lines of approximately 7.5 µm thickness or strictly parallel thin lines oriented perpendicular to the cutting direction—which is in parallel to the blade (Figure 5E,F).

In 359 HREM data sets (59.1% total; 58.2% KO; 61.1% WT) clearly visible parallel lines or plots of broader crests crossed the original HREM images (Figure 5G). They were arranged in the cutting direction—which is perpendicular to the blade. These lines were of variable thickness and most clearly visible in areas containing homogenous tissues, such as the brain and liver.

All data sets showed so-called bleeding through artefacts [24] of various degrees. They were caused by structures beneath the block surface shining through the resin (Figure 5H).

## 4. Discussion

High resolution episcopic microscopy has become increasingly popular over the last decade, as it provides a fast and reliable way of generating digital volume data of organic materials in a very high spatial resolution. In order to assist scientists in the correct interpretation of HREM data, in optimizing specimen and data generation and in identifying abnormalities in mutant mouse embryos, we systematically explored the spectrum of artefacts inherent to HREM data. For this, we relied on more than 600 data sets of mouse embryos, which were produced in a period of approximately five years by scientists and technicians having experienced at least several years of training. The artefacts were grouped according to their most likely causality.

### 4.1. Artefacts Caused by Specimen Harvesting and Manipulation

Like all histological techniques, HREM is an ex vivo method, which visualizes materials extracted from their natural environment [13,25,26,27]. Harvesting and manipulating the specimens causes artefacts that are already known from traditional histology. However, HREM data permit examining them in three-dimensions.

Our results show that, in delicate specimens such as embryos, surface damages are quite common. These damages result from touching the specimens during harvesting, processing and embedding. Despite being handled by experienced scientists, over 50% of our data sets featured ruptures of the skin.

In addition, more than 5% of the data generated in our study showed defects of the skin, which were associated with focal defects of internal tissues. As damaged tissues can also resemble pathologies, the combination of rendered 3D models and virtual 2D resections is crucial for the analysis of specimens, e.g., the ruptured body wall in Figure 2E might be mistaken for a closure defect when only assessed in the 3D model.

Of great importance are our findings that demonstrate that 5% of the examined embryo specimens had perfectly intact surfaces but ruptured internal structures, e.g., the intestine and neural tube were defective, although the embryo skin was perfectly intact. We consider the different elasticity of the fixed tissues to be responsible for that. At any rate, the awareness of the mere existence of such unexpected defects is crucial for being able to correctly distinguish artificial organ defects from true pathologies.

Statistical analyses showed a significant association of all types of tissue damages with the genotype with mutant embryos being affected more often compared to the wildtype embryos. As the mutant embryos were generally smaller in size due to developmental delay caused by the gene knockout, this further underlines the fragility of, especially younger, embryonic specimens. In return, damaging the tissue when collecting specimens of adult tissues such as skin or organ biopsies, is expected to play only a minor role.

The relatively high rate of surface defects in our data also shows the power of the HREM technique. Many of the small damages might only have been detected in the systematic screening of high resolution 3D data in the scope of the DMDD project and otherwise would have remained unnoticed.

Obviously, the frequency of such defects can be reduced by making sure that the specimens are manipulated with extreme care, but depending on the specimens might not be eliminated completely.

### 4.2. Artefacts Caused by Specimen Processing

HREM is a method which creates digital volume data from resin embedded organic materials. Hence, specimen processing is very similar to specimen processing for conventional microscopy. HREM data therefore show a number of artefacts, well known from traditional histology.

The artefacts most relevant for correct data interpretation are tissue shrinkages introduced during fixation, dehydration and infiltration [12,28,29]. Each material reacts slightly differently. Therefore, biological specimens, composed of various tissues, show inhomogeneous shrinkages. Their magnitude does not only correspond with the specimen’s composition, but also with fixation and dehydration protocols. This is especially true for the time period the materials are immersed in the various solutions and slight variations may have dramatic effects.

In general, loose and watery tissues or tissues from which fat is washed out by dehydration shrink much stronger than dense and compact tissues. This becomes obvious in the relation of the epithelial layers of the skin and the subcutis (Figure 4B) and has dramatic effects on the diagnosis of pathologies. Since the thickness of the subcutis of wildtype embryos varies dramatically, it is virtually impossible to feel certain in making the diagnosis of subcutaneous edema in HREM data of mutants. This was already noticed in the DMDD project where the diagnosis “subcutaneous edema” (MP:0013848) exclusively relied on observational data obtained directly after embryo harvesting [17]. Interestingly, statistics showed that extensive wrinkles of the skin (>150 µm deep) were significantly related to the genotype, with the wildtype embryos being more affected. However, as some knockout embryos show a delayed development, the slightly smaller size of those specimens might cause the KO group to be underrepresented.

Relying on the vast experience from traditional histology, rugose skin, displacement and wrinkles of surfaces (diaphragm, pericardium, etc.) and distorted shapes of thin-walled structures, such as veins and the cardiac atria, are easily interpreted as artefacts. Yet, if veins are compressed to slits it is often difficult to distinguish an artificial compression from a stenosis or agenesis. Likewise, it is often complicated to distinguish pathologies of the atrium septum, oval foramen, or Eustachian valve from distortion artefacts resulting from shrinkages of the atrium wall (Figure 4D–H). Here, the 3D information of HREM data greatly assists in the distinction.

Especially in the nervous system, shrinkage artefacts often mimic pathologies. Prominent examples are the quite frequently occurring indentation of the optic cup, which closely resembles coloboma and the arrangement of the spinal ganglia. In coronal and sagittal sections, subsequent spinal ganglia often appear as if touching. Ganglion material which is connected in such a way resembles a pathology termed “fused dorsal root ganglion” (MP:0000963). Our comparisons between wildtype and mutants revealed a way of distinguishing pathology and artefact. Playing on the strength of HREM in visualizing small details in three-dimension, we discovered that in wildtypes, the dorsal root fibers of subsequent spinal nerves, and their ganglia, are almost fully separated. This is independent from the position of the ganglia or their “pseudofusion”. Mingling fibers solely occur in individuals with truly fused ganglion material (Figure 4K).

Other important shrinkage artefacts, that are associated with the CNS, are the detachment of white and grey matter in the forebrain and the presence of cavities surrounding the intracerebral blood vessels. These artefacts must be distinguished from pathologies associated with a spongy appearance of CNS tissues. The same is true for the small vacuoles preferably located in the caudal segments of the spinal cord and cavities distributed in other organs and body parts which might be mistaken for signs of spongiform diseases [12]. We assume these artefacts to be introduced by prolonged fixation periods.

HREM generates volume data by detecting fluorescence signals emitted from the surface of resin blocks. The resin has strong autofluorescence and the specimens embedded in the resin are whole mount stained with eosin to provide unspecific tissue contrasts. Unfortunately, whole mount staining of organic material is not trivial. It requires the staining agent to penetrate all tissues of an intact specimen and to stain the specimen periphery in similar intensity as its core. Especially dense tissues hinder the diffusion of dyes. Therefore, the center of voluminous and dense tissue samples is often improperly stained. An example is the dermis in the center of skin biopsies [6]. In E14.5 embryos, this effect becomes obvious in the center of the liver. In the samples we examined, it remained poorly contrasted in 20% of the specimens. This artefact substantially hindered the semiautomated generation of 3D surface and volume models and the diagnosis of pathologies of the liver sinusoids. Fortunately, protocols to reduce this problem were developed for human tissue samples [6]. They essentially suggest prolonged dehydration and infiltration periods and are capable of substantially improving dye diffusion. Making use of these protocols for processing mouse embryos is possible and can be recommended.

Strikingly, all embryos had small lumps of tissue debris inside their brain ventricles and body cavities. We consider those as pieces of tissues liberated from the ventricle walls during specimen manipulation. In addition, there was a gelatinous substance in the brain ventricles. This has to be kept in mind, when considering the presence of exudates due to infections and abnormal fluid compositions in mutants and diseased individuals.

During specimen preparation and embedding the materials are transferred between dehydration and infiltration solutions and from the infiltration solution into embedding molds. During these transfers, air might become trapped by irregularly shaped protrusions of the surface of the specimens. We hypothesize that, during the exothermic polymerization of the resin, this air then expands and creates hollow spaces in the embedding medium. In embryo specimens, this can create empty cavities, preferably near the limbs, physiologic umbilical hernia and tail. Making sure to transfer specimens entirely covered in solutions eliminates this problem.

Specimen manipulation during infiltration and embedding might result in compressions and distortion of whole specimens. Forty-seven embryos examined for this study showed either bilateral or craniocaudal compression and all embryos showed a strange lordotic deformation of the caudal spine. The latter, we assume, were caused by the manipulations to ensure fixation of the position of the embryos while the resin starts hardening in the embedding molds. Again, it is vital for the correct interpretation of the phenotype of mutants to be aware of these and similar artefacts and distinguish the lordotic distortion of the caudal spine from true pathologies.

### 4.3. Artefacts Caused by Data Generation

HREM data essentially comprise a series of images captured from the subsequently exposed surfaces of a resin block during its sectioning on a microtome. Therefore, HREM images, in contrast to images captured from histological sections, do not exhibit affine and non-affine distortions, introduced by sectioning and section processing. However, there are other types of sectioning artefacts, which result from using resin as an embedding medium and pushing it forcefully against the blade of a knife, or vice versa.

Sectioning on an HREM apparatus might introduce two types of artefacts: firstly, regular lines that are directed in parallel, and secondly, irregular lines or crests that are directed perpendicular to the edge of the blade of the HREM apparatus. The first result from sectioning blocks which were not allowed to harden properly before starting sectioning or later softening due to humidity. Allowing blocks to harden for a few weeks, baking them at approximately 90 °C prior to sectioning or storing them with desiccant almost entirely eliminates this type of artefact. The lines or crests directed perpendicular to the edge of the blade are the result of microscopic imperfections of the knife’s edge. With an increasing number of sections cut, the intensity of these artefacts increases. This reflects the abrasion of the blade’s edge. More than 50% of our data were affected. Per se, they do not resemble pathologies. Yet, they might alter the block face information and thus hinder the diagnosis of small structural pathologies. Their intensity can be significantly reduced, if specially hardened and strongly fixed, non-disposable blades are used for sectioning and the position of the blade is changed after each resin block.

HREM images are precisely aligned, although deviations in the range of 1–2 μm in cutting direction might occur. To minimize such deviations, the HREM apparatus has to be set up in a stable environment. Abnormally positioned sequences of subsequent images, as we detected in six HREM data sets (1%), are unusual. They result from accidentally touching the HREM apparatus or its moving components during the sectioning process.

As a result of imaging freshly exposed surfaces of a resin block, HREM data show so-called bleeding through artefacts [24]. Shining monochromatic light to the block excites the resin and material exposed on the very surface, but also resin and materials located immediately below the surface. Occasionally, this might go down to 10–15 µm, although the intensity significantly decreases with each micrometer. The consequence of this is that block face images add information stemming from materials located beneath the surface to that coming from the surface. In the original HREM images, bleeding through artefacts become apparent as slightly blurred borders of membranes and low contrasts of lumina of capillaries and small tubular structures. In virtual resections cutting sagittally or coronally through HREM data, they also become obvious at surfaces and borders of cavities (Figure 5H). There, they seem to cause fading, conical extensions of intensely stained structures. The tips of the cones are the first trace of a dense structure located relatively deep beneath the block surface. The intensity of the structure grows stronger with each section bringing it nearer to the surface. In addition, the dimension of the plot steadily increases in the subsequently captured images until the structure itself is exposed on the very block surface. After removing the structure with the next section no trace of it remains, leaving a sharp and distinct border to the underlying materials. Bleeding through artefacts can be minimized by exactly following the protocols for mixing the infiltration and embedding solutions and by observing the already published protocols for block processing [2,9]. However, this artefact cannot be fully eliminated. Therefore, it is absolutely essential for all users of HREM to correctly judge the impact of this artefact.

### 4.4. Data Interpretation

In the context of large-scale phenotyping programs like DMDD it is important to compare mutants with reference data based on large numbers of normal individuals of the same developmental stage [20]. This allows for compensating low phenotypic penetrance, anatomic norm-variations and artificial changes of organs, tissues and cells [23]. As embryos are harvested and processed batch-wise according to identical protocols, it is to be expected that all embryos of a single batch show similar artefacts. We therefore recommend using embryos processed in different batches to reduce the influence of artefacts especially introduced during fixation, dehydration, infiltration and embedding.

Proper interpretation of comparative analyses of mutants and reference embryos are not trivial. They need to be performed by clinicians and basic scientists of their respective fields who are experienced in imaging, anatomy, morphology and embryo development. Many studies, especially large-scale phenotyping programs, often produce only small numbers of KO embryos. This bears the problem that low penetrating or subtle defects are easily missed [30]. Defining the full spectrum of pathologies therefore requires breeding and analyzing multiple batches comprised of large numbers of mutants. Even with this, the detection of pathologies remains highly complex.

Artefacts as identified in this study also occur when processing and imaging specimens with alternative 3D imaging techniques. All methods based on harvested or sacrificed specimens such as µMRI, µCT, X-ray phase contrast imaging, OPT or ultramicroscopy, for example, face harvesting and tissue processing artefacts [31]. Techniques based on physical sections such as EMAC, transmission electron microscopy or confocal microscopy even show additional artefacts. They are introduced during collecting and processing single sections which causes non-affine distortions and, in the worst case, loss of data [14,32].

## 5. Conclusions

HREM is a powerful ex vivo method for generating high resolution digital volume data. Like all data generation modalities, it introduces characteristic artefacts, which we systematically evaluated and characterized. These characterizations are essential for the correct interpretation of HREM data and for diagnosing pathologies. In particular, the in-depth presentation and evaluation of the artefacts, which are inherent to HREM data of mouse embryos, are essential for the correct diagnosis of malformations and structural defects in genetically engineered individuals.

## Data Availability

The image data that were the basis of this study are openly available via the homepage of the DMDD project (dmdd.org.uk) hosted by the Francis Crick Institute.

## Supplementary Materials

The following are available online at https://www.mdpi.com/article/10.3390/biomedicines9111711/s1, Table S1: Complete list of statistically analyzed artefacts.
