# Supplementary material for: Artefacts in Volume Data Generated with High Resolution Episcopic Microscopy (HREM)
Source: Biomedicines. 2021 Nov 18;9(11):1711. doi: 10.3390/biomedicines9111711 (PMC8615656; doi:10.3390/biomedicines9111711)
Supplement: Supplementary file 1 [file biomedicines-09-01711-s001.zip › biomedicines-1443410-supplementary.pdf]

Table S1: Complete list of statistically analyzed artefacts. Artefacts grouped according to causality. P-value of statistical difference between knock-out and wildtype. *: p<0.05

Causal group	Artefact		Total (%) n = 607	Knockout (%) n = 409	Wildtype (%) n = 198	p-value
Specimen harvesting						
	Surface damage		359 (59.1)	255 (62.3)	104 (52.5)	.021 *
	incl.	head	84 (13.8)	63 (15.4)	21 (10.6)	.109
		limb	152 (25)	108 (26.4)	44 (22.2)	.265
		body wall	56 (9.2)	39 (9.5)	17 (8.6)	.705
	Additional tissue damage		41 (6.8)	34 (8.3)	7 (3.5)	.028 *
	Umbilical hernia damage		192 (31.6)	142 (34.7)	50 (25.3)	.019 *
	Additional intestine damage		38 (6.3)	32 (7.8)	6 (3.0)	.022 *
	Removed umbilical hernia		6 (1.0)	6 (1.5)	0 (0.0)	.087
	Blood around embryo		34 (5.6)	21 (5.1)	13 (6.6)	.472
	Tissue w/o surface damage		34 (5.6)	28 (6.8)	6 (3.0)	.055
	Extensive blood inside heart		528 (87.0)	354 (86.6)	174 (87.9)	.649
	incl.	50-75%	348 (57.3)	239 (58.4)	109 (55.1)	.429
		>75%	180 (29.7)	115 (28.1)	65 (32.8)	.234
Specimen processing and embedding						
	Vacuoles		105 (17.3)	77 (18.8)	28 (14.1)	.153
	incl.	extensive	11 (1.8)	10 (2.4)	1 (0.5)	.093
		sparsely	25 (4.1)	14 (3.4)	11 (5.6)	.215
		only caudal CNS	69 (11.4)	53 (13.0)	16 (8.1)	.076
	Low contrast inside liver		131 (21.6)	79 (19.3)	52 (26.3)	.051
	Deformation		47 (7.7)	29 (7.1)	18 (9.1)	.387
	incl.	cranio-caudal	11 (1.8)	7 (1.7)	4 (2.0)	.789
		lateral	36 (5.9)	22 (5.4)	14 (7.1)	.408
	Cavities		19 (3.1)	15 (3.7)	4 (2.0)	.275
	incl.	inside specimen	12 (2.0)	10 (2.4)	2 (1.0)	.234
		outside specimen	5 (0.8)	3 (0.7)	2 (1.0)	.724
		inside + outside	2 (0.3)	2 (0.5)	0 (0.0)	.324

	Tilt coronal/sagittal >5°		131 (21.6)	83 (20.3)	48 (24.2)	.268
	incl.	>10°	91 (15.0)	59 (14.4)	32 (16.2)	.574
	Rotation >5°		36 (5.9)	23 (5.6)	13 (6.6)	.645
	incl.	>10°	26 (4.3)	14 (3.4)	12 (6.1)	.132
	Extensive skin wrinkles		98 (16.1)	56 (13.7)	42 (21.2)	.018 *
	Ap-shrunk atrium		54 (8.9)	39 (9.5)	15 (7.6)	.427
	Mucosa separated (pars pylorica)		163 (26.9)	106 (25.9)	57 (28.8)	.454
	incl.	mucosa separated (stomach)	48 (7.9)	29 (7.1)	19 (9.6)	.284
Data generation						
	Broken block		4 (0.7)	1 (0.2)	3 (1.5)	.070
	Block face obscured		4 (0.7)	1 (0.2)	3 (1.5)	.070
	Data incomplete		12 (2.0)	8 (2.0)	4 (2.0)	.958
	incl.	top	6 (1.0)	5 (1.2)	1 (0.5)	.402
		middle	1 (0.2)	1 (0.2)	0 (0.0)	.486
		bottom	4 (0.7)	1 (0.2)	3 (1.5)	.070
		out of FOV	1 (0.2)	1 (0.2)	0 (0.0)	.486
	Misaligned sections		6 (1.0)	3 (0.7)	3 (1.5)	.361
	Changing contrast		15 (2.5)	7 (1.7)	8 (4.0)	.083
	Out of focus		71 (11.7)	53 (13.0)	18 (9.1)	.165
	Sectioning scratches		359 (59.1)	238 (58.2)	121 (61.1)	.493
	Resin hardening		84 (13.8)	54 (13.2)	30 (15.2)	.515
